# Synergistic Antimycobacterial Actions of *Knowltonia vesicatoria* (L.f) Sims

**DOI:** 10.1155/2012/808979

**Published:** 2012-05-07

**Authors:** Antoinette Labuschagné, Ahmed A. Hussein, Benjamín Rodríguez, Namrita Lall

**Affiliations:** ^1^Department of Plant Science, University of Pretoria, Gauteng Pretoria 0002, South Africa; ^2^Department of Chemistry of Medicinal Plants, National Research Center, El-Tahrir Street, Dokki, Cairo 12311, Egypt; ^3^Instituto de Química Orgánica, Consejo Superior de Investigaciones Científicas (CSIC), Juan de la Cierva 3, 28006 Madrid, Spain

## Abstract

*Euclea natalensis* A.DC., *Knowltonia vesicatoria* (L.f) Sims, and *Pelargonium sidoides* DC. are South African plants traditionally used to treat tuberculosis. Extracts from these plants were used in combination with isoniazid (INH) to investigate the possibility of synergy with respect to antimycobacterial activity. The ethanol extract of *K. vesicatoria* was subjected to fractionation to identify the active compounds. The activity of the *Knowltonia* extract remained superior to the fractions with a minimum inhibitory concentration (MIC) of 625.0 **μ**g/mL against *Mycobacterium smegmatis* and an MIC of 50.00 **μ**g/mL against *M. tuberculosis*. The *K. vesicatoria* extract was tested against two different drug-resistant strains of *M. tuberculosis*, which resulted in an MIC of 50.00 **μ**g/mL on both strains. The combination of *K. vesicatoria* with INH exhibited the best synergistic antimycobacterial activity with a fractional inhibitory concentration index of 0.25 (a combined concentration of 6.28 **μ**g/mL). A fifty percent inhibitory concentration of this combination against U937 cells was 121.0 **μ**g/mL. Two compounds, stigmasta-5,23-dien-3-ol (1) and 5-(hydroxymethyl)furan-2(5H)-one (2), were isolated from *K. vesicatoria* as the first report of isolation for both compounds from this plant and the first report of antimycobacterial activity. Compound (1) was active against drug-sensitive *M. tuberculosis* with an MIC of 50.00 **μ**g/mL.

## 1. Introduction

For the last 40 years there has been little progress in the treatment of Tuberculosis (TB). The standard albeit dated treatment regime is strict and lengthy (6–9 months) resulting in adverse side effects and inevitable patient noncompliance. It is no surprise that the emergence of multiple and extensively drug resistant strains of *Mycobacterium tuberculosis* (*M. tb*) is on the rise. The WHO recently reported that in some areas of the world, one in four people with TB becomes ill with a form of the disease that can no longer be treated with standard drugs [1]. In addition, HIV/AIDS increases the risk for developing active TB and renders TB difficult to diagnose and treat. The TB-HIV/AIDS coinfection rate in South Africa is distressingly high, with an estimated 73 percent of new TB patients coinfected with HIV [2]. The search for new TB treatments that are effective against resistant strains of *M. tb* and treatments which can augment the potential of existing drugs against the disease is more important today than at any other time in history. Without the introduction of new treatments, TB patients will run out of options for effective drugs.

Plant products have received considerable attention as potential anti-TB agents with a recent review emphasizing plant products as sources of antimycobacterial extracts and compounds [3]. Most traditionally used plant therapies rely for their effects on a variety of compounds and synergy between these compounds, however, there are numerous benefits for isolating and identifying active constituents from these bioactive plants. These benefits include characterising toxicity profiles, simpler determination of modes of action, and new activities of known compound which adds to the wealth of information on phytochemicals. Combining plant extracts and current TB drugs holds advantages such as decreased toxicity profiles, increased bioavailability and activity, and reduced onset of microbial resistance. Testing isolated compounds from plant extracts, using whole extracts alone, and in combination with other extracts and current anti-TB drugs, covers a wider range for activity and possible treatment therapies. In this study the synergistic antimycobacterial activity of three South African plants as well as their cytotoxicity and the isolation, identification, and antimycobacterial activity of compounds obtained from the ethanol (EtOH) extract of *Knowltonia vesicatoria *is reported.

In previous experiments, the EtOH extract of *K. vesicatoria *(aerial parts) was found to be active against both *Mycobacterium smegmatis* and *M. tuberculosis* [4]. *Knowltonia vesicatoria* (Ranunculaceae) is relatively new to the field of tuberculosis research, and in general very little work has been done on this specific plant, even though traditional uses related to TB have been documented [5]. Recently the genus *Knowltonia* has been subsumed within the genus *Anemone,* and *K. vesicatoria* is now known as *Anemone vesicatoria *(L.f) Prantl [6]. To avoid confusion the name *Knowltonia vesicatoria* has been retained throughout the paper. One of the objectives of the study was to identify the antimycobacterial compounds present in the *K. vesicatoria* extract and to eliminate the possibility of tannins as the antimycobacterial actives. In addition, extracts prepared from two well-known South African plants, *Pelargonium sidoides* DC. (root, EtOH) and *Euclea natalensis* A.DC. (root, chloroform (CHCl_3_)) were included in the study to investigate the possibility of synergistic antimycobacterial action when combined with each other, *K. vesicatoria*, and the first-line antitubercular drug, isoniazid (INH).

*Pelargonium* species (Geraniaceae) are highly valued by traditional healers for their curative properties and are wellknown for treatment of coughs, diarrhoea, and tuberculosis. A review by Brendler and van Wyk [7] discusses all aspects of *Pelargonium sidoides*, including a complete and comprehensive take on the anti-TB, antibacterial, antifungal and immunomodulating activity of this valuable plant.

The roots of *Euclea *species (Ebenaceae) are used in southern African traditional medicinal preparations to treat chest complaints, chronic asthma, leprosy, and infections, among other ailments [8–11]. *Euclea natalensis* is a familiar plant to TB research and the antimycobacterial activity against *M. tb *by extracts and compounds of *Euclea natalensis* roots has been previously reported [12], including the synergistic activity of this extract with INH [13].

## 2. Materials and Methods

### 2.1. Plant Material

*Knowltonia vesicatoria* syn *Anemone vesicatoria *was procured in the province of Gauteng, South Africa. The stems and leaves (aerial parts) were collected during January. Previously, roots of *P. sidoides* were collected from the Free State province, and roots from *E. natalensis* were collected from KwaZulu-Natal province of South Africa. A voucher specimen (PRU 096449, P 092559 and N.L. 22, resp.) for each plant was deposited and identified at the H.G.W.J. Schweickerdt Herbarium (PRU), University of Pretoria, South Africa.

### 2.2. Microorganisms and Cell Lines

*Mycobacterium smegmatis *(MC^2^ 155) cultures, obtained from, American Type Culture Collection (ATCC), Culture Collection (ATCC), MD, USA were kindly donated from the Medical Research Council (MRC) in Pretoria, South Africa. The cultures were kept on Middlebrook 7H11 agar (Sigma-Aldrich Chemical Co., South Africa) and stored at approximately 8°C, for no longer than one month. Several aliquots of the prepared mycobacterial cultures in Middlebrook 7H9 broth (Sigma-Aldrich Chemical Co., South Africa) were frozen in cryovials at −70°C.

Three *M*. *tb *strains were used in the experimental procedures, which were carried out at the Medical Research Council (MRC), Pretoria, South Africa. H37Rv (ATCC 27294), a drug-susceptible strain of *M. tb*, sensitive to the first-line antituberculous drugs, INH, rifampicin (RIF), ethambutol (EMB), and streptomycin (STR), was obtained from the ATCC. *Mycobacterium tuberculosis *were plated onto slants of Löwenstein-Jensen (LJ) medium and allowed to grow for 3-4 weeks at 37°C. Two clinical drug resistant strains, 4388 (resistant to INH and EMB) and 5497 (resistant to RIF, INH, EMB, STR) were maintained in BACTEC 12B medium (7H12 medium containing ^14^C-labeled substrate, palmitic acid) at 37°C until a growth index (GI) of 400 was reached. Both resistant strains were obtained from an MRC proficiency test (round 14) from Belgium. These resistant strains were only used to test the activity of the *K. vesicatoria *(EtOH) extract.

The histiocytic lymphoma cell line, U937, was obtained from Highveld Biological (Pty) (Ltd.) (Sandringham, South Africa) and maintained in complete RPMI 1640 medium (pH 7.2) (Sigma-Aldrich Chemical Co., South Africa), supplemented with 10% heat-inactivated foetal calf serum (FCS), 2 mM L-glutamine, and a 0.1% antimicrobial solution (pennicilin, streptomycin, and an antifungal. fungizone).

### 2.3. Extraction and Isolation

Prepared extracts of *Euclea natalensis* (CHCl_3_) and *Pelargonium sidoides* (EtOH) were kindly donated by Professor N. Lall. The collected aerial parts of *K. vesicatoria* were allowed to air-dry in open sample bags away from direct sunlight. The dried plant material (530.0 g) was extracted with 2.50 L of 100% EtOH. The solvent and plant material was placed on an electric shaker and vigorously shaken by hand twice a day for seven days. Each day the plant material were filtered, the extract concentrated under reduced pressure with a rotary evaporator, and clean EtOH was added to the remaining plant material. A total yield of 148.4 g of dried extract was obtained (28% of the total dried plant material). Sixty grams of the *K. vesicatoria* EtOH extract was subjected to a solvent partitioning method for the removal of polyphenolics [14]. The dried EtOH extract was dissolved in 1.5 L of a 9 : 1 EtOH : dH_2_O solution which was then partitioned with an equal volume of hexane. The hexane layer was concentrated under reduced pressure and stored for further testing of the nonpolar components (2.35 g). A portion of the 90% EtOH layer was concentrated under reduced pressure and stored for further testing (11.88 g). The remaining EtOH layer (750 mL) was partitioned with equal volumes of CHCl_3_ : methanole (MeOH) (4 : 1) and water. The two layers were then separated. The EtOH layer was concentrated under reduced pressure and stored (13.27 g). The chloroformic layer was then washed with an equal volume (750 mL) of 1% w/v NaCl in water. The chloroformic layer was then dried under reduced pressure, yielding 9.86 g of polyphenol-free extract. All the layers were screened against *M. smegmatis* and the active layers tested against *M. tb*.

The crude *K. vesicatoria* extract (28.00 g) was subjected to silica column chromatography (CC, size 10 × 23 cm) using hexane: ethyl acetate (EtOAc) mixtures of increasing polarity (0 to 100%) followed by MeOH. Fractions (1–26) of 500 mL were collected. Similar fractions were pooled together and dried, which resulted in nine main fractions. These nine fractions were subjected to a TLC bioautographic antibacterial assay using *M. smegmatis*. Fraction 3 (F3, 404.0 mg) was subjected to silica gel CC (size 3 × 25 cm) gradient elution from hexane to ethyl acetate (0 to 60%) on the basis of its inhibitory activity on *M. smegmatis, *to yield pure compound (1) (30.70 mg, Figure 2). Fraction 8 (F8, 378.9 mg) from the first column contained very few constituents with one main compound and was chosen for further isolation with a silica gel CC (hexane-ethyl acetate, 30 to 100%). Fifty subfractions were obtained and pooled together as two main fractions (F8.1 and F8.2). Fraction 8.2 was further purified with a Sephadex column using MeOH which resulted in the 3 : 2 isomer mixture of (2) and its enantiomer (3) (123.3 mg, Figure 2). The structural identity of the mixture was elucidated by physical (mp. [*α*]_D_) and spectroscopic (^1^H and ^13^C NMR) data and was also subjected to 2D COSY, HMQC, HMBC and NOESY spectra. The isomer mixture (30.00 mg) was subjected to column chromatography (CC, 3 × 30 cm) using 150 g of inactivated silica (1.00 mL d H_2_O for each 10.00 g of silica) eluted with hexane :  EtOAc (40 to 60%). This procedure separated a portion (8.00 mg) of the main isomer (2) from the mixture. Isolation of minor isomer (3) was unsuccessful.

### 2.4. Antimycobacterial Activity of Solvent Partitioned Fractions on *M. smegmatis* Using Microplate Susceptibility Testing

Cryopreserved or freshly scraped colonies were suspended in fresh Middlebrook 7H9 broth (Sigma-Aldrich Chemical Co., South Africa) and incubated for 24 h at 37°C. The overnight liquid culture was then transferred to a sterile test tube containing 20–25 glass beads (with a 2.00 mm diameter) and homogenised by using a vortex mixer (Heidolph, Germany) for 5–10 min. The broth culture was then left still for 5–10 min to let larger clumps of mycobacteria settle. The supernatant was carefully decanted to a sterile flask and adjusted with Middlebrook 7H9 broth base to an optical density of 0.2 (log phase) at 550 nm (Beckman DU-720 UV spectrophotometer), yielding 1.26 × 10^8^ colony-forming units per millilitre (CFU/mL). The microdilution test was performed in 96-well microtiter plates as described earlier [15, 16].

The plant extract and fractions were dissolved in 10% dimethyl sulfoxide (DMSO) to obtain a stock concentration of 10 mg/mL. Ciprofloxacin (Sigma-Aldrich Chemical Co., South Africa) at final concentrations ranging from 2.00 × 10^−3^ to 7.80 × 10^−5^ mg/mL served as the positive drug control [17]. Serial twofold dilutions of each test sample were made with Middlebrook 7H9 broth base (Sigma-Aldrich Chemical Co., South Africa) to yield volumes of 100 mL/well with final concentrations ranging from 2.50 to 0.08 mg/mL. *Mycobacterium smegmatis* (100 mL adjusted to an optical density value of 0.2 to ensure the bacteria suspension was at the start of the log phase and approximately 1.26 × 10^8^ CFU/mL upon test commencement) was also added to each well containing the samples and mixed thoroughly to give a final volume of 200 mL/well. The solvent control, DMSO at 12.50%, did not show inhibition on the growth of the bacteria. Tests were done in triplicate on two different occasions.

The plates were sealed with parafilm and incubated at 37°C for 24 h. The minimum inhibitory concentration (MIC) of samples was detected following addition (40 *μ*L) of 0.20 mg/mL p-iodonitrotetrazolium chloride (INT, Sigma-Aldrich Chemical Co., South Africa) to duplicates of each sample triplicate and incubated at 37°C for 30 min [16]. Viable bacteria reduced the yellow dye to a pink colour. The MIC was defined as the lowest sample concentration that prevented this change and exhibited complete inhibition of bacterial growth. The minimal bactericidal concentration (MBC) was determined by taking 50.0 *μ*L aliquots from the remaining INT excluded wells to 150.0 *μ*L of 7H9 broth in a fresh 96-well plate. This new plate was sealed with parafilm and incubated for 48 h at 37°C. The MBC was recorded as the lowest concentration of sample which did not produce the pink colour change after the addition of 40.0 *μ*L INT (0.20 mg/mL).

### 2.5. Antitubercular Rapid Radiometric Assay Using *M. tuberculosis*

The radiometric respiratory techniques using the BACTEC 460 system (Becton Dickinson Diagnostic Instrument, Sparks, MD, USA) were used for susceptibility testing against all the *M. tb* strains as described previously [18].

Solutions of all the test samples were prepared in DMSO to obtain their respective concentrations. Previous results for *K. vesicatoria *show an MIC of 50.00 *μ*g/mL against H37Rv [4], and was tested here at three concentrations (100.0, 50.00, and 25.00 *μ*g/mL) in triplicate. This extract was also tested against two drug-resistant strains of *M. tb *at the same concentrations. According to Lall et al. [12] the chloroform root extract of *E. natalensis *has an MIC of 8.00 *μ*g/mL against susceptible *M. tb*. This extract was tested again at concentrations ranging from 16.00, 8.00 to 4.00 *μ*g/mL in triplicate. The EtOH extract of *P. sidoides *exhibited an MIC above 5000 *μ*g/mL [19] and was tested at three concentrations (10000, 5000, and 2500 *μ*g/mL) in triplicate. The hexane fraction (F2) and the tannin-free fraction (F4) obtained from the tannin clean-up partitioning were tested in triplicate at three concentrations (100.0, 50.00, and 25.00 *μ*g/mL) based on the antimycobacterial activity of these samples against *M. smegmatis*. Compounds (1) and (2) were tested in triplicate at four concentrations (200, 100, 50, and 25.00 *μ*g/mL). All the samples were dissolved in 100% DMSO to obtain stock concentrations that were subsequently serially diluted twofold to give the three test concentrations. None of the samples exceeded a final DMSO concentration above 1.0% as control experiments showed that a final concentration of DMSO (1.0%) in the medium had no adverse effect on the growth of *M. tb*. The primary drug INH (Sigma-Aldrich Chemical Co., South Africa), at a concentration of 0.02 mg/mL, served as the drugcontrol in the bioassay.

### 2.6. Determining Synergistic Antimycobacterial Activity on *M. tuberculosis* Using the BACTEC Radiometric Assay

The activity of three different drug, and extract combinations was evaluated at sub-MIC levels (below original MIC values) so that each component (extract or drug) was present at concentrations corresponding to 1/2, 1/4, and 1/8 of the MIC. Analysis of the drug combination data was achieved by calculating the fractional inhibitory concentration (FIC) index [20] with a general equation for use with any number (*n*) of drugs in a combination as follows: FIC = (MIC_*a*  combination_/ MIC_*a*  alone_) + (MIC_*b*  combination_ + MIC_*b* alone_) +*⋯*+ (MIC_*n*  combination_ + MIC_*n*  alone_). The subscripts represent the different components in the drug combination. The FIC was interpreted as follows: FIC ≤ 0.5, synergistic activity; FIC = 1, indifference/additive activity; FIC ≥ 2 or more, antagonistic activity [13, 21].

Three different combinations of plant extracts and INH were tested for possible synergistic activity against the drug susceptible *M. tb* strain. Combination 1 (C1) included the extracts of *E. natalensis* (CHCl_3_), *K. vesicatoria* (EtOH), and *P. sidoides* (EtOH) in a four-drug combination with the first-line drug INH. Combination 2 (C2) only included the three plant extracts in a three-drug combination. Combination 3 (C3) combined the *K. vesicatoria* (EtOH) extract and INH in a two-drug combination. The BACTEC radiometric method, as described before [13], was used to determine the synergistic activity of these different amalgamations.

### 2.7. Cytotoxicity

**Figure 1:**
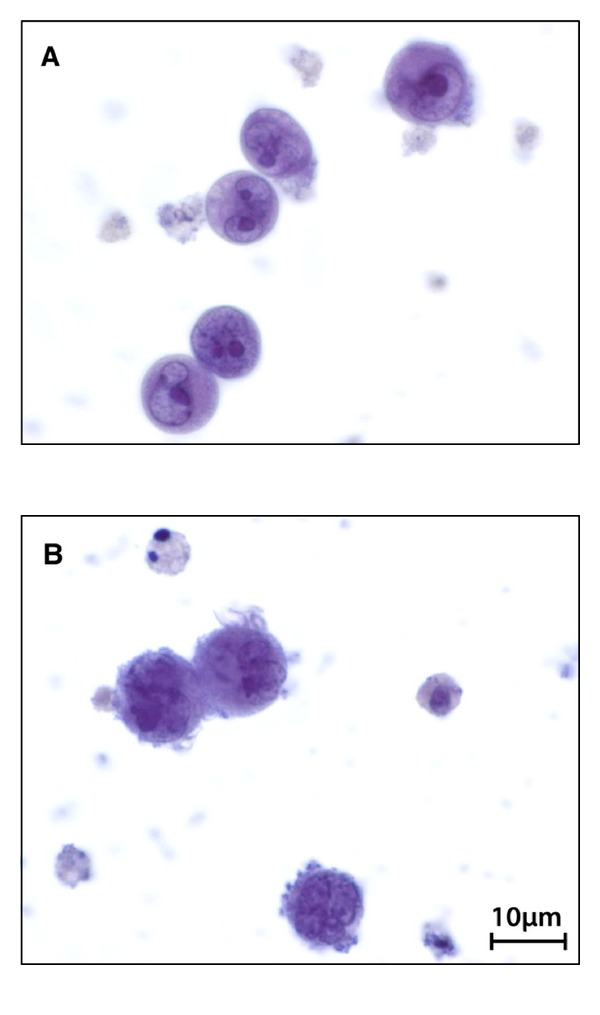
Undifferentiated (A) and differentiated (B) U937 cells as viewed under a light microscope (100x magnification).

All reagents were procured from Highveld Biological (Pty) (Ltd.) (Sandringham, South Africa) unless indicated otherwise. The U937 cells were grown to a density of 5 × 10^8^ cells/mL, centrifuged, and washed with phosphate buffered saline (PBS) solution. The concentration of cells was adjusted to 1 × 10^5^ cells/mL in complete medium containing a final concentration of 0.10 *μ*g/mL phorbol 12-myristate 13-acetate (PMA) (Sigma-Aldrich Chemical Co., South Africa). Two hundred microlitres of the cell suspension were seeded into the inner wells of a 96-well tissue culture plate, while the outer wells received 200.0 *μ*L of incomplete medium. The cells were incubated for 24 hours at 37°C in an atmosphere of 5% CO_2_ to induce differentiation of monocytes to mature macrophages (Figure 1) [22, 23]. Cytotoxicity was measured by the 2,3-bis(2-methoxy-4-nitro-5-sulfophenyl)-5-[(phenylamino)carbonyl]-2-*H*-tetrazolium hydroxide (XTT) method using the Cell Proliferation Kit II (Roche Diagnostics GmbH). Dilution series of *E. natalensis*,* K*. *vesicatoria *(EtOH crude and tannin-free), and *P. sidoides* extracts were prepared at various concentrations (400.0 to 3.125 *μ*g/mL). Synergistic combinations were tested at MIC and sub-MIC levels to maintain the correct ratios of each extract as tested against *M. tb*, accordingly each combination was tested at different starting concentrations. Combination 1 was made up to a combined concentration of 5058.2 *μ*g/mL in the first well with a final concentration of 39.52 *μ*g/mL. Combination 2 was made up to a combined concentration of 5058.0 *μ*g/mL in the first well with a final concentration of 39.52 *μ*g/mL and C3 with concentrations ranging from 50.20 to 0.392 *μ*g/mL. The pure compounds (INH, (1) and (2)) were made up to a stock concentration of 20.00 mg/mL and serially diluted to start with a concentration of 200.0 to 1.562 *μ*g/mL from the first wells to the last in the microtitre plates. The isomer mixture (compounds (2) and (3)) was treated in the same way. These dilutions were added to the inner wells of the microtiter plate and incubated for 72 h. After 72 h, 50.0 *μ*L of XTT reagent (1.0 mg/mL XTT with 0.383 mg/mL PBS) was added to the wells and the plates were then incubated for 1-2 hours. The positive drug, (Actinomycin D, Sigma), at a final concentration range of 5.0 × 10^−2^ to 3.9 × 10^−4^ *μ*g/mL, was included. After incubation, the absorbance of the colour complex was spectrophotometrically quantified using an ELISA plate reader (PowerWave XS, Bio-Tek), which measures the OD at 450 nm with a reference wavelength of 690 nm. DMSO (0.04%) was added to serve as the control for cell survival. GraphPad Prism 4.03 software was used to statistically analyse the 50% inhibitory concentration (IC_50_) values.

## 3. Results and Discussion

### 3.1. Identification of the Isolated Compounds

**Compound (1):**

**Figure 2:**
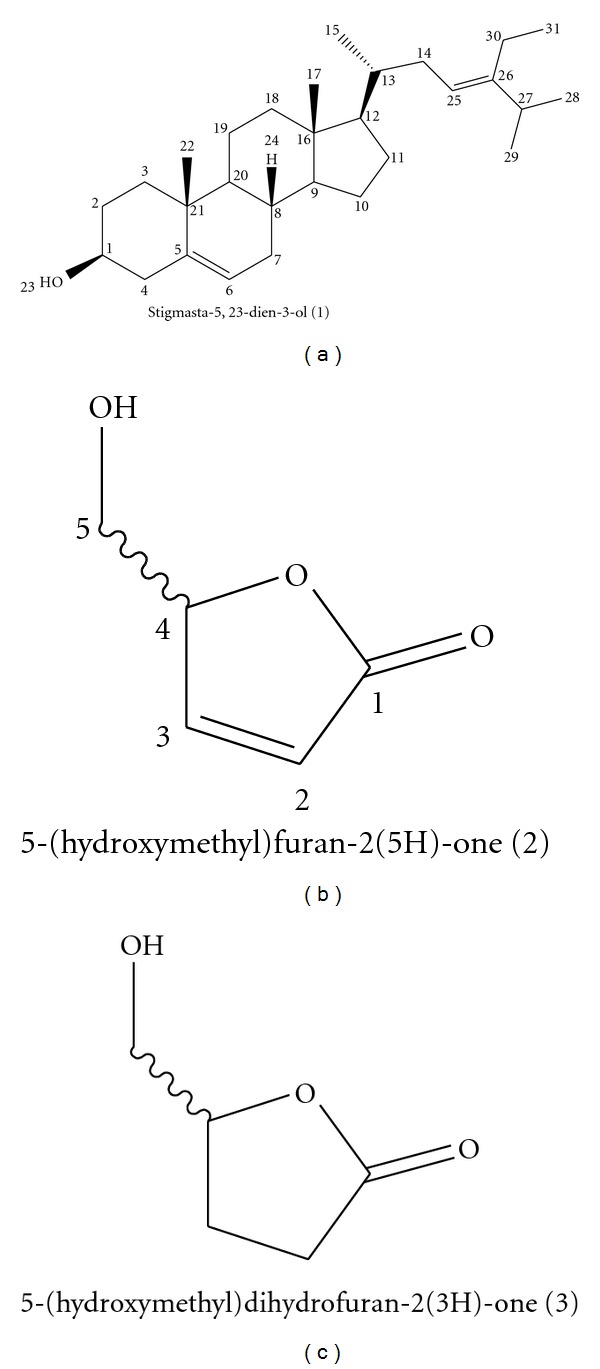
Chemical structures of isolated compounds from *Knowltonia vesicatoria*.

*Stigmasta-5,23-dien-3-ol*. (Figure 2) was obtained as clear crystals and was identified based on NMR data (^1^H and ^13^C) which were compared with those reported in literature [24, 25].

**Isomer mixture:**

5-(hydroxymethyl)furan-2(5H)-one and 5-(hydroxymethyl)dihydrofuran-2(3H)-one. (Figure 2) was obtained as a yellow oil. This sample is a ≈ 3 : 2 mixture of compounds (2) and (3), respectively, as was revealed by the integral of the ^1^H NMR spectrum and also by the absence of HMBC and COSY correlations between the signals corresponding to each one of the constituents.

**Compound (2):**

5-(hydroxymethyl)furan-2(5H)-one. (Figure 2) [**α**]_D_-5.7 (c 0.16, CHCl_2_); ^1^H NMR (500 MHz, CDCl_3_): **δ** 7.47 (1H, dd, J_3,2  _= 5.8 Hz, J_3,4_ = 1.6 Hz, H-3), 6.21 (1H, dd, *J*_2,3_ = 5.8 Hz, *J*_2,4_ = 2.1 Hz, H-2), 5.15 (1H, dddd, *J*_4,2_ = 2.1 Hz, *J*_4,3_ = 1.6 Hz, *J*_4,5A  _= 3.9 Hz, *J*_4,5B  _= 5.1 Hz, H-4), 4.03 (1H, dd, *J*_5A,5B  _= 12.2 Hz, *J*_5A,4  _ = 3.9 Hz, H_A_-5), 3.79 (1H, dd, *J*_5A,5B  _ = 12.2 Hz, *J*_5b,4  _= 5.1 Hz, H_B_-5). The absolute stereochemistry at the C-4 asymmetric centre not determined. Since (2) was obtained as a yellow oily substance, the undefined stereochemistry at the C-4 asymmetric centre is most likely the (*S*)- and not the (*R*)-form as the latter crystallises and the (*S*)-form is an oil [26].

### 3.2. Microdilution Assay Using *M. smegmatis*

Although studies have shown that *M. smegmatis *could be more resistant to drug activity than *M. tuberculosis* [27–29], screening samples against nonpathogenic *M. smegmatis* gives quick results in any laboratory fitted for microbial tests and provides a good indication of which samples will also be active against *M. tuberculosis*. In most cases this strategy saves both time and expensive reagents necessary to test numerous samples against the pathogen, *M. tb*. In this case, the purpose of screening the partitioned fractions was to promptly establish if the tannin-free extract still exhibits the same activity as the tannin-containing extract and whether the tannin-concentrated fraction had higher antimycobacterial activity than the whole extract. Results clearly indicate that this is not the case (Table 1).

The tannin-free extract (F4) exhibited an MIC fourfold higher (2500 *μ*g/mL) than that of the crude tannin containing EtOH extract (625.0 *μ*g/mL). This does not automatically point to the conclusion that tannins are the responsible components for *Knowltonia*'s antimycobacterial activity. None of the partitioned fractions from *K. vesicatoria* exhibited an MIC or MBC as low as that of the crude or “whole” EtOH extract. Fraction 1, which still contained all of the polyphenolic compounds but no nonpolar compounds, had the same activity profile as that of F4, with an MIC of 2500 *μ*g/mL and an MBC value of more than 2500 *μ*g/mL. The fractions with concentrated levels of tannins, fractions 3 (containing mostly polyphenols) and 5 (existing mainly of NaCl and polyphenol residue), had no activity at the highest concentration tested (2500 *μ*g/mL) and were subsequently not included for the *M. tb* antimycobacterial test. The highest activity seen for the fractions was that of the nonpolar compounds (F2) with an MIC and MBC of 1250 and 2500 *μ*g/mL, respectively. Fraction 2 and the tannin-free fraction (F4) were tested against *M. tb*. The positive drug control, ciprofloxacin, exhibited an MIC equal to its MBC value of 1.250 *μ*g/mL, which is comparable to its mycobactericidal value found in the literature [30]. The highest percentage DMSO used for the samples in the assay (2.50%) did not inhibit *M. smegmatis* growth as evident with the MIC/MBC value of 12.50%. Taken as a whole, the results imply that either the active compounds were damaged during the partitioning procedure or that synergy within the *K. vesicatoria* extract itself forms the basis of its antimycobacterial activity.

### 3.3. Antitubercular Rapid Radiometric Assay Using *M. tuberculosis*

The antimycobacterial assays of the extracts against *M*. *tuberculosis* using the BACTEC radiometric method showed that *K*. *vesicatoria* inhibited *M*. *tuberculosis* at an MIC of 50.0 *μ*g/mL. *Pelargonium sidoides *and *E. natalensis* inhibited the bacteria at the 5000 *μ*g/mL and 8.00 *μ*g/mL against *M*. *tuberculosis* (Table 2). These results correspond very well with previous MIC values obtained for all of the extracts [4, 11, 12, 18, 19]. The sensitive strain of *M. tb* was not susceptible to either F2 (hexane layer) or F4 (tannin-free extract) obtained from the* K*. *vesicatoria* tannin clean-up procedure at the highest concentration tested (100 *μ*g/mL). The antituberculosis positive drug, INH, inhibited the growth of *M*. *tuberculosis *at 0.2 *μ*g/mL during all the independent assays.

The combination drug action showed that only C1 and C3 exhibited synergistic antimycobacterial activity. Although the combined MIC of C1 (*E. natalensis* (CHCl_3_) + *K. vesicatoria* (EtOH) + *P. sidoides* (EtOH) + INH) was reduced eightfold from 5058.2 to 632.30 *μ*g/mL, the FIC (0.5) indicates that the combination had threshold synergistic activity. This is mainly due to the high ratio of *P. sidoides* (5000 *μ*g/mL equal to 98.8%) present in the combination. The same situation, where the *P. sidoides* extract overwhelms the other components in the amalgamation, is seen with C2 (*E. natalensis* (CHCl_3_) + *K. vesicatoria* (EtOH) + *P. sidoides* (EtOH)), which is slightly reflected by the FIC (1.5), indicating an additive effect of the combination. The best synergistic result with an FIC of 0.25 was seen for C3 (*K. vesicatoria* (EtOH) + INH) where the combined MIC is reduced eightfold, from 50.20 to 6.275 *μ*g/mL. This synergistic activity of C3 implies that if a patient were to take INH prescribed for the treatment of TB and combined this regime with the extract of *K. vesicatoria,* other possible benefits such as reduced length of INH treatment and decreased toxicity due to lower intake concentrations of *K. vesicatoria* could subsist. Testing the cytotoxicity of this combination is the next step to shed some light on this possibility.

The isomer mixture exhibited no inhibitory activity against *M. tb* at the highest concentration tested (200.0 *μ*g/mL). Since lactones are known to exhibit significant antibacterial properties, it was interesting to note that in this case the sterol was considerably more active than the lactone with an MIC of 50.00 *μ*g/mL compared to the lactone MIC of 200.0 *μ*g/mL. It has been reported that phytosterols have anti-inflammatory, antibacterial, antifungal, antiulcerative, antioxidant, and antitumoral activities [31, 32]. Additionally, Saludes et al. [33] reported the antimycobacterial activity of five phytosterols isolated from *Morinda citrifolia* against *M. tb*. Three of these isolated sterols closely resemble the structure of *stigmasta-5,23-dien-3-ol*, with the only differences being the position and degree of saturation. Stigmasta-4-en-3-one and stigmasta-4,22-dien-3-one (used in combination) had an MIC lower than 2.00 *μ*g/mL; stigmasterol had an MIC of 32.00 *μ*g/mL. Another phytosterol saringosterol, isolated from *Lessonia nigrescens*, (a brown algae) exhibited extremely low toxicity compared to its MIC of 0.250 *μ*g/mL [34].

*Knowltonia vesicatoria* inhibited both drug-resistant strains of *M*. *tuberculosis* at an MIC of 50.0 *μ*g/mL (Table 2). This activity indicates a mechanism of action different to INH, EMB, STR, and RIF. These drugs target cell wall synthesis (INH, EMB), inhibit gene transcription (RIF), and inhibit protein synthesis (STR) [35]. The antimycobacterial mechanism of action of *K. vesicatoria* has to be investigated in order to confirm this supposition as a different mechanism of action to INH could hold positive implications on preventing drug resistance as well as targeting strains already resistant to INH with the use of a drug therapy that combines *K. vesicatoria* and INH.

### 3.4. Cytotoxicity

The XTT cytotoxicity assay is a rapid and cost-effective tool to help choose optimal candidates, those samples with low cytotoxicity and high antimycobacterial activity, similar to a therapeutic dose, and to exclude any samples too toxic to test at their antimycobacterial concentration for ensuing intracellular assays. The results obtained (Table 2) indicated that the cytotoxicity effects of the four plant extracts on U937 cells demonstrated marginal toxicity except for *E*. *natalensis*, which showed high toxicity at a fifty percent inhibitory concentration (IC_50_) of 12.22 *μ*g/mL against the macrophages. The tannin-free extract of *K*. *vesicatoria* (F4) had the highest IC_0050_ value (64.77 *μ*g/mL) compared to the other plant extracts; the lowered toxicity is most likely due to the lack of the protein binding polyphenols. *Knowltonia vesicatoria *and* P. sidoides* showed similar toxicity exhibiting IC_50_ values at 41.25 and 43.54 *μ*g/mL, respectively (Table 2). With the synergistic combinations, the approximate IC_50_ values for combinations 1 and 2 were calculated as 100.3 and 108.6 *μ*g/mL, respectively. Synergistic combination C3 had a resultant IC_50_ of 121.70 *μ*g/mL. The toxicity profiles of the combinations were much better than the individual extracts indicating a synergistic action on lowering cytotoxicity.

The pure compounds, which included the antitubercular drug INH and the cytotoxic drug Actinomycin D, showed very different cytotoxic profiles compared to the extracts and combinations. With the compounds isolated from *K. vesicatoria*, compound (1) was more than twofold as toxic to the cells when compared to compound (2), with an IC_50_ of 16.41 and 44.70 *μ*g/mL, respectively. The 3 : 2 isomer mixture of *5-(hydroxymethyl)furan-2(5*H*)-one *(2) and* 5-(hydroxymethyl)dihydrofuran-2(3*H*)-one* (3) was not toxic to the macrophages even at the highest concentration tested (200.0 *μ*g/mL) with more than 50 percent of the cells still viable at this concentration. Isoniazid had a similar effect on the cells, with 80 percent of the cells still viable at 200.0 *μ*g/mL, indicating an IC_50_ above this value. Actinomycin D, which served as the positive control in the cytotoxicity assay, exhibited an IC_50_ of 3.80 × 10^−3^ *μ*g/mL.

Similar to a therapeutic index, it is necessary to compare the difference between cytotoxicity and antimycobacterial activity of the tested samples in order to choose the best candidates for possible treatment options. Of all the samples INH had the broadest difference in toxicity when compared to biological activity, followed by C3, *E. natalensis*, the isomer mixture and the EtOH extract of *K. vesicatoria*. *Pelargonium sidoides* had the narrowest therapeutic range followed by F4, C2, C1, and compounds (1) and (2).

## 4. Conclusion

This study adds to current literature by demonstrating the synergistic antimycobacterial activity of the crude EtOH extract from the aerial parts of *K. vesicatoria* in its entirety and in a drug combination with the first-line drug, isoniazid. Based on these findings we assume that a drug combination of INH with *K. vesicatoria* could help prevent and combat-resistant strains; however, further studies will have to be carried out in order to demonstrate its efficacy.

This paper is the first to report on the isolation of *stigmasta-5, 23-dien-3-ol,* and *5-(hydroxymethyl)furan-2(5*H*)-one* from *K. vesicatoria*; it is also the first report of the antimycobacterial activity of both these compounds. Subsequent and ongoing work includes investigating the possible mechanism of action, intracellular antimycobacterial activity, and measuring the immunomodulation of the most active candidates.

The various aspects of the challenges faced in TB drug discovery are applicable to other infectious agents. With *Knowltonia vesicatoria* being reintroduced to the field of medicinal plant science, its extracts and isolated compounds could also be applied to screening for activity against other pathogens. It is the interplay of codeveloped methods on the natural products chemical perspective that will improve the chances of treatment success.

## Figures and Tables

**Table 1:** MIC and MBC values of the solvent-partitioned fractions against *M. smegmatis* compared to the crude EtOH extract of *K. vesicatoria*.

Sample	MIC^a^ (*μ*g/mL)	MBC^b^ (*μ*g/mL)
*K. vesicatoria* (EtOH)	625.0	1250
F1	2500	>2500
F2 (hexane layer)	1250	2500
F3	>2500	>2500
F4 (tannin-free)	2500	>2500
F5	>2500	>2500
DMSO%	12.50	12.50
Ciprofloxacin	1.250	1.250

^a^Minimum inhibitory concentration.

^b^Minimum bactericidal concentration.

**Table 2:** Antimycobacterial activity against *M. tuberculosis* and cytotoxicity on U937 cells of test samples.

Samples	MIC^a^ (*μ*g/mL)	FIC^b^	ΔGI_4-3_ ± SD^c^	IC_50_ (*μ*g/mL ± SD)^d^
V2^e^	—	—	22.0 ± 2.94	—
INH (0.2 *μ*g/mL)	0.20	—	−2.50 ± 2.12	>200.0
Actinomycin D^f^	NT^g^	—	—	3.820 ± 0.258 ×10^−3^
*K. vesicatoria* EtOH	50.00^h^	—	−6.00 ± 0.58	41.25 ± 0.205
F4^i^	>100.0	—	275.5 ± 45.3	64.77 ± 1.812
F2^j^	>100.0	—	288.5 ± 65.1	NT
*E. natalensis* CHCl_3_	8.000	—	−3.50 ± 0.88	12.22 ± 0.025
*P. sidoides* EtOH	5000	—	20.00 ± 1.85	43.54 ± 0.465
Compound (1)	50.00	—	−9.00 ± 1.41	16.41 ± 0.135
Compound (2)	100.0^k^	—	—	44.70 ± 0.50
Isomer mixture (2) and (3)	>100.0	—	117.0 ± 8.49	>200.0
C1	1.000/6.250/625.0/0.025^l^	0.50	−5.50 ± 3.53	100.3 ± 2.450
C2	4.000/25.00/2500^m^	1.50	17.00 ± 5.65	108.6 ± 0.89
C3	6.250/0.025^n^	0.25	−1.5 ± 0.707	121.7 ± 2.079

^a^Minimum inhibitory concentration.

^b^Fractional inhibitory concentration index.

^c^ΔGI value (mean ± standard deviation).

^d^Fifty percent inhibitory concentration.

^e^10^−2^ inoculum control.

^f^Cytotoxicity assay positive control.

^g^Not tested.

^h^MIC value for *K. vesicatoria* against both clinical drug-resistant *M. tb* strains: 4388 (resistant to INH and ETH; V2 ΔGI_4-3_ ± SD = 32.5 ± 4.95) and 5479 (resistant to INH, EMB, STR, RIF; V2 ΔGI_3-2_ ± SD = 34.5 ± 0.71) was found to be 50.00 *μ*g/mL with ΔGI_4-3_ = 2.0 ± 4.24 and ΔGI_3-2_ = 0.33 ± 0.57, respectively.

^i^Fraction 4: tannin-free *Knowltonia* fraction.

^j^ Fraction 2: hexane *Knowltonia* fraction.

^k^BACTEC MIC not determined, MIC determined via microplate assay.

^l^Eightfold reduction of respective MIC values for *E. natalensis* (CHCl_3_) + *K. vesicatoria* (EtOH) + *P. sidoides* (EtOH) + INH.

^m^Twofold reduction of respective MIC values for *E. natalensis* (CHCl_3_) + *K. vesicatoria* (EtOH) + *P. sidoides* (EtOH).

^n^Eightfold reduction of respective MIC values for *K. vesicatoria* (EtOH) + INH.
